# Targeting protein modification: a new direction for immunotherapy of pancreatic cancer

**DOI:** 10.7150/ijbs.101861

**Published:** 2025-01-01

**Authors:** Xinyu Ge, Ke Zhang, Jie Zhu, Yuan Chen, Zhengwang Wang, Peng Wang, Peng Xu, Jie Yao

**Affiliations:** 1 Department of Hepatobiliary and Pancreatic Surgery, Northern Jiangsu People's Hospital Affiliated to Yangzhou University, Jiangsu 225000, China.; 2 The Yangzhou School of Clinical Medicine of Dalian Medical University, Jiangsu 225000, China.

**Keywords:** Post-translational modifications, Immunotherapy, Targeted therapy, Immune escape, Pancreatic cancer

## Abstract

Post-translational modifications (PTMs) alter protein conformation by covalently attaching functional groups to substrates, influencing their biological activity, mechanisms of action, and functional performance. PTMs and their interactions are essential to many critical signal transduction processes, including tumor transformation, cancer progression, and metastasis in pancreatic cancer. Additionally, advancements in tumor immunotherapy indicate that PTMs are essential in immune cell activation, transport, and energy metabolism. This study aimed to investigate the effects of different PTMs on immunotherapy for pancreatic cancer, providing new perspectives and suggesting directions for future research.

## Introduction

Pancreatic cancer predominantly comprises pancreatic ductal adenocarcinoma (PDAC), a malignancy associated with poor prognosis 1. In 2022, approximately 510,566 new cancer cases were reported globally, resulting in 467,005 deaths, which makes it the sixth most common cause of cancer-related deaths globally 2. The 5-year survival rate for patients with stage I or II PDAC after surgery is 24.6% 3. Despite the advancements in treatments, including chemotherapy, neoadjuvant, and targeted therapies, significant challenges persist 4.

Immunotherapy is a new and effective treatment option 5. It primarily includes immune checkpoint inhibitors (ICIs), adoptive cell therapy (ACT), and monoclonal antibody therapy 6. ACT is a highly personalized treatment that targets cancer through the transplantation of autologous or allogeneic tumor-specific T cells 7. Significant advancements have been made in adoptive cell therapies, including Chimeric Antigen Receptor T-Cell Immunotherapy (CAR-T) and T-cell receptor-engineered T cell (TCR-T) cell therapies 8, 9. Monoclonal antibody is a type of targeted therapy characterized by significant specificity, extended serum half-life, strong binding affinity, and ability to activate immune effector functions 10.

Despite significant advancements in immunotherapy, the near-universal resistance of PDAC to immunotherapy is a significant exception in human cancers. Effective responses are observed in less than 1% of patients with microsatellite instability-high (MSI-H) tumors. The tumor microenvironment (TME) of PDAC is frequently described as "cold," marked by the limited presence of effector T cells and a significant influx of myeloid cells 11, 12. Furthermore, features including a low mutational burden and an immunosuppressive TME hinder T cell activation, migration, and functionality, thereby exacerbating the challenges to adaptive immunity in PDAC 13, 14. The most promising strategy for PDAC treatment entails comprehensive study and optimization of immunotherapy, shifting focus from solely targeting malignant cell proliferation and invasion to investigating the complex interactions between tumors and TME.

**Figure 1:**
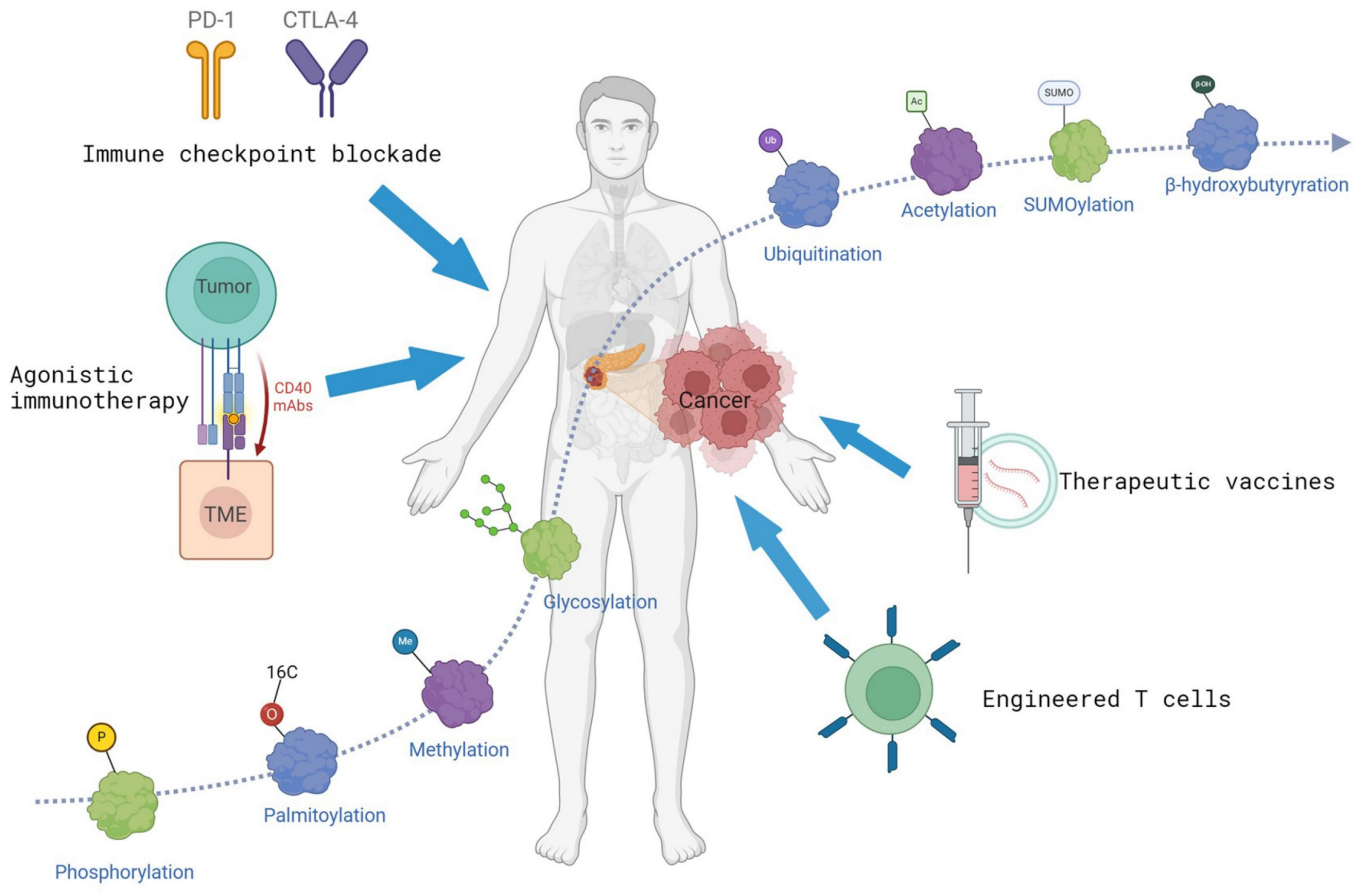
PTMs in immunotherapy of pancreatic cancer. Many proteins and PTMs (such as phosphorylation, acetylation, ubiquitination, etc.) are implicated in tumorigenesis. PTMs can influence the efficacy of immunotherapy. The figure is generated with BioRender (https://biorender.com).

Post-translational modifications (PTMs) influence the complexity and diversity of the proteome by covalently binding functional groups to substrate proteins. The method involves adding various functional groups into the side chains of amino acids, including acetyl, phosphate, sugar, and methyl groups. The polypeptide chain undergoes multiple PTMs within different cellular compartments, including the nucleus, cytosol, endoplasmic reticulum, and Golgi apparatus 15. During physiologic and pathologic conditions, it can enhance the functional diversity of proteins by modulating protein folding, activity, stability, localization, signal transduction, and binding 16, 17. Consequently, these modifications are essential in various physiological activities, including signal transduction, gene expression, and cell cycle regulation 18, 19. With the enhanced accessibility of genomic sequencing data and the rapid development of detection methods, over 600 types of PTMs have been discovered to date 20. The most common include protein phosphorylation, acetylation, SUMOylation, glycosylation, and palmitoylation (Fig. 1).

PTMs are essential in immune activity in the body, significantly influencing immune cell activation, signal regulation, immune response, and tumor metabolic reprogramming. They regulate TME by affecting immune cell differentiation and function 21-25. PTMs can directly or indirectly affect the efficacy of immunotherapy by regulating immune checkpoints or altering the TME 26. PTMs can regulate the immunogenic characteristics of cancer cells, affecting their recognition and susceptibility to immune system attacks 24.

Because protein PTMs regulate cancer development and progression, examining these alterations in the context of immune responses may offer a comprehensive understanding of the mechanisms regulating interactions between cancer cells and immune cells. Herein, we systematically examine and present the recent advancements regarding the role of PTMs in the immunotherapy of PDAC.

## Phosphorylation

Phosphorylation is a classic and reversible PTM prevalent in eukaryotes. In mammals, approximately 30% of proteins undergo phosphorylation, a process dynamically regulated by protein kinases and phosphatases 27. This alteration is essential for numerous cellular activities, including cell division, membrane transport, gene expression regulation, and protein interactions 28. Numerous phosphorylation events have been identified in PDAC (Table 1). Furthermore, phosphorylation is essential in tumor immunotherapy.

A primary challenge in tumor immunotherapy research is investigating the immune evasion mechanisms associated with immune checkpoints. According to their targets, immune checkpoint inhibitors include Programmed Cell Death Protein 1 (PD-1) inhibitors, Programmed Death-Ligand 1 (PD-L1) inhibitors, and Cytotoxic T-lymphocyte associated protein 4 (CTLA-4) inhibitors. The interaction between PD-L1 and PD-1 exerts a negative regulatory effect, facilitating peripheral immune tolerance 29. The regulatory role of the PDCD1 gene is well-established 30; however, PTMs are significant factors affecting PD-1 and PD-L1 interaction. Zhang *et al.* 31 reported that the limited efficacy of immunotherapy in PDAC is primarily due to PD-L1 dephosphorylation by Never in Mitosis A-related kinase 2 (NEK2). NEK2 enhances PD-L1 stability by inhibiting its proteasomal degradation through phosphorylation at T194/T210 residues and further stabilizes PD-L1 through glycosylation at N192, N200, and N219 sites 31, 32.

Gemcitabine (GEM), the most commonly used chemotherapeutic agent for PDAC, induces Signal Transducer and Activator of Transcription 1 (STAT1) phosphorylation after treatment and elicits various PD-L1-inducing cytokines, including IFN-γ, IL-6, and TNF-α 33. A previous study reported that statins combined with GEM inhibit STAT1 phosphorylation, significantly reducing PD-L1 expression and enhancing CD8+ T cell infiltration 34. Moreover, statins reduce YAP/TAZ expression through AKT phosphorylation, further inhibiting PD-L1 expression 35. In TME, neutrophil extracellular traps induce T cell exhaustion and dysfunction via PD-L1, a mechanism closely linked to the chemotactic effect of CXC motif chemokine receptor 2 (CXCR2) on neutrophils 36-38, a mechanism closely linked to the chemotactic effect of CXCR2 on neutrophils, inhibits the recruitment and function of CXCR2 in neutrophils by inducing STAT1 dephosphorylation at Tyr701 in these cells. In animal models, Nifurtimox significantly enhances PDAC sensitivity to GEM and PD-1 blockade therapy 39, 40. Zhang *et al.* 41 reported that Polo-like Kinase 1 (Plk1) induces retinoblastoma protein (RB) phosphorylation at S758, leading to dysregulated NF-κB translocation and increased PD-L1 expression. Inhibition of Plk1 enhances sensitivity to immune checkpoint blockade. NSG3, a vesicular transport protein, is a potential diagnostic and prognostic marker that inhibits PDAC cell proliferation and invasion and suppresses Erk1/2 phosphorylation, thereby inhibiting PD-L1 expression and improving immunotherapy outcomes 42, 43.

T cells are essential in protecting the body from pathogens. Enhancing T-cell infiltration can significantly improve the efficacy of existing cancer immunotherapies, including ICB therapy 44, 45. T-cell activity is regulated by the phosphorylation of specific proteins or enzymes within the tumor. In patients with PDAC, the inhibition of IRAK4 phosphorylation in tumor cells downregulates Hyaluronan synthase 2 (HAS2) through an NF-κB-dependent mechanism. This reduction in HAS2 levels mitigates T-cell exhaustion and enhances responsiveness to checkpoint immunotherapies, including anti-CTLA-4 and anti-PD-1 46, 47. The T cell receptor (TCR) is activated by phosphorylation at Tyr-323 (pY323), which binds to p38-activated MAPK as an alternative pathway for p38 activation. TCR-mediated activation of CD4+ tumor-infiltrating lymphocytes (TILs) leads to alternative p38 activation and pro-inflammatory cytokine production 48. Targeting this alternative p38 pathway in T cells demonstrates promising preventive and therapeutic effects in PDAC models by disrupting downstream pro-inflammatory pathways 49. Interleukin-35 (IL-35), a cytokine of the IL-12 family primarily produced by CD4+ T cells and B cells, induces STAT3 phosphorylation. This process inhibits CD8+ T-cell infiltration and activation and promotes tumor growth 50, 51. Targeting IL-35 to enhance T-cell infiltration and transform the TME of PDAC from "cold" to "hot" is an effective strategy to improve the efficacy of immunotherapy. Chemotherapy should address immune suppression within tumors to achieve adequate therapeutic outcomes. PX-478, for example, can inhibit HIF-1α expression, increase eIF2α phosphorylation levels, enhance GEM immunogenicity, and strengthen cytotoxic T-cell responses against PDAC cells 52.

The immune response in PDAC is partially regulated by immunosuppressive myeloid cells, rendering these cells a promising target for immunotherapy 53. Macrophages are among the most abundant immune cell types in the TME and facilitate tumor progression by creating an immunosuppressive TME from the early stages 54, 55. Re-polarizing tumor-associated macrophages (TAMs) toward an M1-like phenotype has been proposed as a potential therapeutic option for cancer 56. Dual-specificity tyrosine-regulated kinase 1B (DYRK1B), a kinase that regulates tyrosine phosphorylation, is present in 90% of pancreatic cancer cases and is negatively correlated with macrophages in tumor tissues 57, 58. Inhibiting DYRK1B in TAMs accelerates their polarization toward an M1 phenotype, thereby reducing cancer cell surface marker CD24 expression 59. This, subsequently, enhances immune cell identification and eradication of cancer cells 58. STAT3 is essential in solid tumor progression 60, 61. Phosphorylated STAT3, as a direct target of miR-506, reprograms M2-polarized macrophages into an M1 phenotype, thereby reversing the immunosuppressive microenvironment in PDAC.

Natural killer (NK) cells are innate lymphocytes, and their activation is regulated by the interactions between NK receptors and target cells 62, 63. This makes NK cell-based therapies a significant focus of innovation in immunotherapy. Two NK cell subsets can be identified in human peripheral blood: (1) the CD56bright subset, which secretes immunoregulatory cytokines, and (2) the CD56dim subset, comprising approximately 90% of the total number of NK cells, which exerts cytotoxicity through the cell surface Fc receptor CD16 64.

The NF-κB signaling pathway regulates the differentiation of NK cell subsets and their immune responses. Phosphorylation of iκB protein in PDAC cells facilitates nuclear translocation of NF-κB. The activation of NF-κB subsequently promotes CXCL8 and the transcription factor P65 transcription, facilitating the migration of radiation-induced CD56dim NK cells from tumor cells and inhibiting tumor growth. This indicates that combining NK cell adoptive therapy with radiotherapy can effectively induce tumor cell apoptosis 65. A previous study has reported that polysaccharides enhance the antitumor effects of GEM through the activation of NK cells 66. In PDAC, polysaccharides secreted by SEP bind to the TLR4 receptor on NK cells, upregulating ERK, JNK, p38, and NF-κB phosphorylation levels. TLR4/MAPKs/NF-κB signaling pathway activation increases NKG2D expression in NK cells, thereby synergistically enhancing the anti-pancreatic tumor effects of GEM 67. In addition, NK cells can be combined with therapeutic antibodies for cancer treatment 68. Enhancing NK cell FcR effector functions through Interleukin-21 (IL-21) is a promising strategy to improve the efficacy of cetuximab. Following IL-21 interaction with NK cell surface receptors, the STAT1 phosphorylation level increases. When NK cells are stimulated by cetuximab-coated tumor cells, the ERK phosphorylation level increases, leading to intracellular activation of NK cells, reduced tumor burden, and improved therapeutic outcomes 69.

## Glycosylation

Glycosylation is essential for stabilizing membrane protein expression and preserving normal physiological function 70. Eight glycosylation pathways have been identified, with N-glycosylation and O-glycosylation significantly associated with disease progression. PDAC tumors demonstrate distinct alterations in glycosylation (Table 2), including an increased abundance of the sialic acid Lewis A antigen CA19-9 71. Besides, PDACs have elevated levels of fucosylated and branched and truncated O-glycans 72-74, which are associated with tumor progression and poor prognosis (Table 2). Additionally, abnormal glycosylation contributes to tumor immune evasion 75. Consequently, glycosylation is a potential target for anticancer therapy 76.

Sialylation is a pervasive and complex form of glycosylation that has become a target for cancer therapy because of its immunosuppressive properties 77. In PDAC, the elevated ST3Gal1 and ST3Gal14 expression results in increased α2,3-sialylation on tumor cells, facilitating the differentiation of monocytes into immunosuppressive TAMs by binding to myeloid cell receptors Siglec-7 and -9 75. Furthermore, excessive tumor sialylation inhibits NK cell activity and disrupts Teff/Treg balance, facilitating immune escape 78. Salivation inhibition enhances the immune response through various mechanisms, including facilitating dendritic cell maturation, increasing the number and activation status of effector immune cells, particularly CD8+ T cells, and enhancing cytotoxic T cell activities 79.

Mucin is an essential defense barrier in the body that is frequently overexpressed and abnormally glycosylated in PDAC, acting as a source of tumor-associated antigens and potential therapeutic targets. Mucin 4 (MUC4) is a compelling TAA. Wei *et al.* 80 first reported that transducing dendritic cells with pan-DR helper T cell epitopes or the universal T epitope (PADRE) with HLA-A1 and HLA-A2-specific MUC4 epitopes led to the upregulation of dendritic cell activation markers including HLA-DR, CD80, and CD86, thereby inducing an effective MUC4-specific cytotoxic T cell response. Furthermore, glycopeptide immunization using glycosylated MUC4 tandem repeat peptides has demonstrated effective antigen-specific immune responses in PDAC mouse models 81. Mucin 1 (MUC1) is a protein composed of repetitive 20-amino-acid sequences and undergoes extensive O-glycosylation. In PDAC, MUC1 glycosylation is irregularly distributed, which enhances the recognition of the immune system and processing of the protein structure, consequently eliciting an immune response against MUC1 81. A phase I/II clinical trial demonstrated that dendritic cell vaccines infused with MUC1 significantly enhanced CD8 and CD4 T cell activities, effectively improving immunosuppression in patients undergoing pancreatic surgery 82.

Engineered T cells expressing chimeric antigen receptors (CARs) signify a promising research focus in immunotherapy 83. However, CAR T therapy encounters challenges, including inefficient delivery and penetration to tumor sites, and its efficacy depends on the density and accessibility of tumor cell antigens 84. Abnormal glycosylation in tumor cells manifests as an extracellular glycan layer on the cell surface. This glycoprotein shell can participate in basic biological processes and disrupt immune responses by masking immune cell epitopes. Glycosyltransferase 5 (MGAT5) is an essential gene that regulates N-glycan chains 85. MGAT5-derived N-glycans provide strong protection against pancreatic cancer 86. In PDAC, defects in N-glycosylation due to MGAT5 knockout induce robust immune synapses between tumor cells and 44v6 CAR T cells. This interaction is characterized by increased F-actin accumulation, enhanced granule convergence, and a reduced distance from the microtubule organizing center to F-actin. In PDAC with N-glycosylation defects, activated 44v6 T cells exhibit enhanced signaling of the calcium-dependent phosphatase nuclear factor and NF-κB, thereby enhancing CAR T cell efficacy in the immune response against PDAC 87. On the other hand, tumor polysaccharide coating can also be used as a marker for immunotherapy 88. For instance, 5E5 CAR T cells specifically target the Tn-MUC1 glycopeptide epitope on PDAC cell surfaces, resulting in significant tumor accumulation and exhibiting significant anticancer efficacy in mouse models 89.

## Acetylation

Acetylation is a dynamic and reversible PTM in which acetyl groups are transferred to substrates by acetyltransferases. However, proteins undergo deacetylation through the action of deacetylases 90 (Table 3). This process, termed histone acetylation, was initially identified in histones 91. In mammals, acetylation occurs on non-histone lysine residues, including those in high mobility group proteins, tubulin, and p53 92. Consequently, the enzymes involved are reclassified as lysine acetyltransferases 93. Acetylation has been implicated in the pathogenic processes of pancreatic cancer development (Table 3). Recent studies highlight the importance of acetylation modifications in immune system function and tumor immunity 94.

Histone deacetylases (HDAC) can remove acetyl groups from acetylated proteins 95. HDACs have garnered attention for their role in immune evasion, making them a promising target for therapeutic strategies 96. Currently, there are five HDAC inhibitors approved for clinical use. These inhibitors can disrupt PD-L1 and PD-1 interaction. HDAC5 inhibits immune responses and increases T regulatory cells, highlighting its significance in antitumor immunity. In PDAC, inhibition of HDAC5 suppresses NF-κB-mediated PD-L1 expression and improves the efficacy of anti-PD-1 therapy. Therefore, HDAC inhibitors can enhance the sensitivity of PDAC to immune checkpoint therapy 97. The HDAC inhibitor LBH589 can enhance histone acetylation in the PD-L1 promoter region, thereby rapidly enhancing PD-L1 expression 98. Moreover, HDAC3 inhibitors, including RGFP966, reduce PD-L1 mRNA and protein levels, thereby enhancing immune surveillance and reversing immune evasion 99. Chin-King Looi *et al.* 100 found that HDAC inhibitors givinostat and dacinosta can reverse the sensitivity of Cytotoxic T lymphocytes (CTLs) resistant PDAC cells to CTLs. Furthermore, HDAC inhibitors can mitigate immune evasion by reprogramming tumor-associated myeloid-derived suppressor cells (MDSCs). Entinostat reprograms MDSCs in pancreatic tumor models, transforming immune-resistant tumors into those responsive to checkpoint therapies 101.

## Ubiquitination

Ubiquitination is the binding of ubiquitin to specific amino acids as monomers or polymers. Ubiquitin-activating enzymes facilitate this process and depend on the synchronized function of three essential proteins: the ubiquitin-activating enzyme (E1), the ubiquitin-conjugating enzyme (E2), and the ubiquitin ligase (E3) 102. E3 ligases are essential for recognizing specific substrate proteins, thereby tightly regulating ubiquitination. Deubiquitinating enzymes (DUBs) reverse the process. Emerging evidence indicates that ubiquitination and deubiquitination play key roles in regulating the progression and prognosis of pancreatic cancer (Table 4). The dynamic equilibrium between ubiquitination and deubiquitination regulates protein expression levels, ensuring protein function stability, which ultimately affects substrate activity 103, 104.

**Figure 2:**
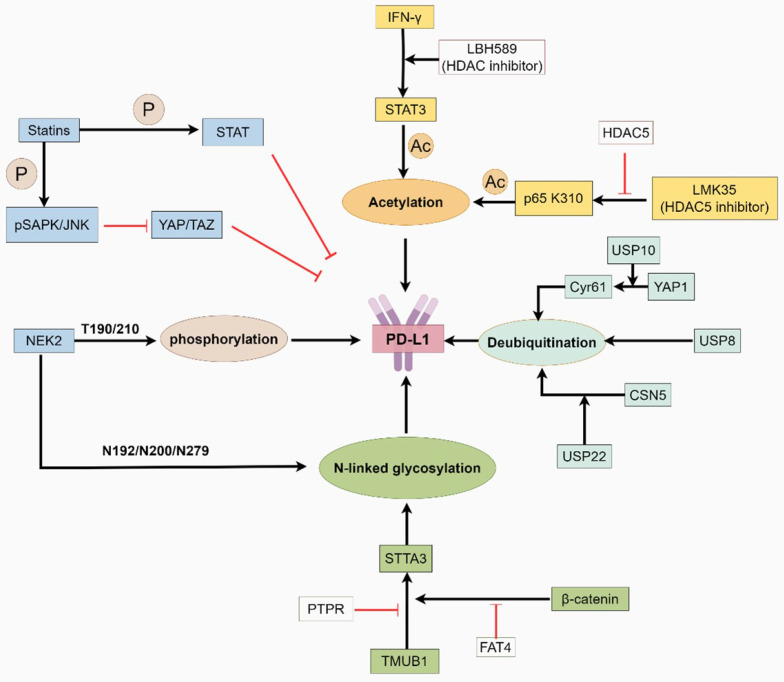
The function of PD-L1 is regulated by post-translational modifications. This graphic was generated using Figdraw.

The half-life of PD-L1 is regulated by ubiquitination and deubiquitination 105. DUBs alter substrate conformation by cleaving ubiquitin moieties, facilitating tumor immune evasion. DUBs regulate PD-L1 deubiquitination through distinct mechanisms. For instance, Ubiquitin-specific peptidase 10 (USP10) is a deubiquitinating enzyme that exhibits oncogenic effects in multiple tumors 106, 107. YAP1, a key effector of the Hippo pathway, is involved in tumorigenesis and immunosuppression 107. USP10 deubiquitinates and stabilizes YAP1/Cyr61, thereby increasing PD-L1 and galectin-9 in the TME and increasing the M2 macrophage proportion. This facilitates tumor invasion and immune evasion 108, 109. In addition, Ubiquitin-specific peptidase 8 (USP8), another deubiquitinase, is associated with T-cell function 110. USP8, a new PD-L1 deubiquitinase, interacts with PD-L1, thereby inhibiting its ubiquitination-dependent proteasomal degradation in pancreatic cancer. USP8 inhibitors combined with anti-PD-L1 therapy stimulate cytotoxic T cells and improve efficacy 111. Ubiquitin-specific peptidase 22 (USP22) is overexpressed in various malignant tumors 112-114. On the one hand, it directly regulates the stability of PD-L1 through ubiquitination. On the other hand, USP22 deubiquitinates COP9 signalosome subunit 5 (CSN5) and regulates PD-L1 protein levels through the USP22-CSN5-PD-L1 axis (Fig. 2) 114. In the USP22 knockdown model, a decrease in M2 macrophage infiltration was also observed, indicating its multiple roles in immune regulation 115.

The E3 ubiquitin ligase determines the specificity of the ubiquitination reaction by identifying the substrate within the ubiquitin-protease system 116. A growing body of research highlights the critical role of E3 ligases in modulating tumor immune responses 137. E3 ubiquitin ligases RNF43 and ZNRF3 function as tumor suppressors in stem cell homeostasis by down-regulating Wnt receptors 117. Single-cell sequencing revealed that RNF43-deficient tumor progression was accompanied by complex Immunological change, demonstrating low myeloid and high lymphocyte TME. The absence of RNF43 may result in the up-regulation of CTLA4 expression, potentially diminishing the efficacy of immunotherapy 118. The linear ubiquitin chain assembly complex (LUBAC) can facilitate tumor progression in the TME 119. NF31 inhibition, as a component of LUBAC, significantly enhances the sensitivity of tumor cells to NK- and T-cell-mediated killing. *In vivo* studies using tumor transplantation models have demonstrated that the impairment of RNF31 function results in diminished tumor growth and enhanced T-cell infiltration and efficacy 120.

## Sumoylation

Sumoylation is a dynamic and reversible PTM 121. Initially discovered in yeast 122. Five SUMO subtypes have been identified in humans 123. The SUMOylation process involves an enzyme cascade comprising the SUMO E1 activating enzyme, E2 conjugating enzyme, E3 ligase, and deSUMOylating enzyme. SUMO-1 modifies substrates as a monomer, whereas SUMO-2/3 can form poly-SUMO chains 124. SUMOylation is essential for regulating cellular functions, including protein activity, subcellular localization, and transcriptional regulation 123.

Emerging evidence highlights the significant role of SUMOylation in pancreatic cancer. SUMOylation affects PDAC adaptation and survival by regulating essential processes, including cell proliferation and migration 125. Alexander Biederstädt *et al.* 126 found an aggressive pancreatic cancer subtype that co-actives MYC and SUMO pathways, which affect prognosis. Increased MYC activity increased PDAC sensitivity to SUMO inhibitors.

Previous studies have reported that mitotic SENP3 activation can lead to micronuclei formation in cancer cells and induce innate immunity through the cGAS-STING signaling pathway, thereby inducing host antitumor immunity 127, 128. Analysis of pancreatic cancer samples from public databases revealed that SUMO1/2 expression is inversely associated with the infiltration of various tumor-infiltrating immune cells, including activated B cells, memory B cells, and effector memory CD8 T cells. This correlation encompasses most immune modulators, including chemokines, MHC molecules, immune promoters, and chemokine receptors 129. Sumit Kumar *et al.* 129 have confirmed these findings. The SUMOylation inhibitor TAK-981 exhibits dual potential in PDAC treatment. It inhibits cell mitosis by targeting the SUMO pathway and simultaneously activates interferon signaling, enhancing CD8 T cells and NK cell infiltration.

## Conclusion and Discussion

PTMs are critical events in signal transduction and are essential for regulating protein conformation, function, movement, and interactions. Beyond the classical PTMs discussed above, emerging modifications, including β-hydroxybutyrylation and lysine crotonylation, have been linked to tumor immune response and metabolism 130, 131.

Crotonylation is a modification that utilizes crotonyl-CoA as the donor 132. In PDAC, crotonylation of metabolic enzymes significantly regulates tumor progression. For example, CBP/p300 facilitates IDH1 crotonylation at Lys224 and Lys236, which impacts metabolic levels. Besides, CBP/p300, combined with HDAC1 and HDAC3, facilitates MTHFD1 decrotonylation at K354 and K553, promoting pancreatic cancer development 133. Investigating the mechanisms and functions of crotonylation in metabolic enzymes during PDAC progression may reveal new therapeutic targets.

**Figure 3:**
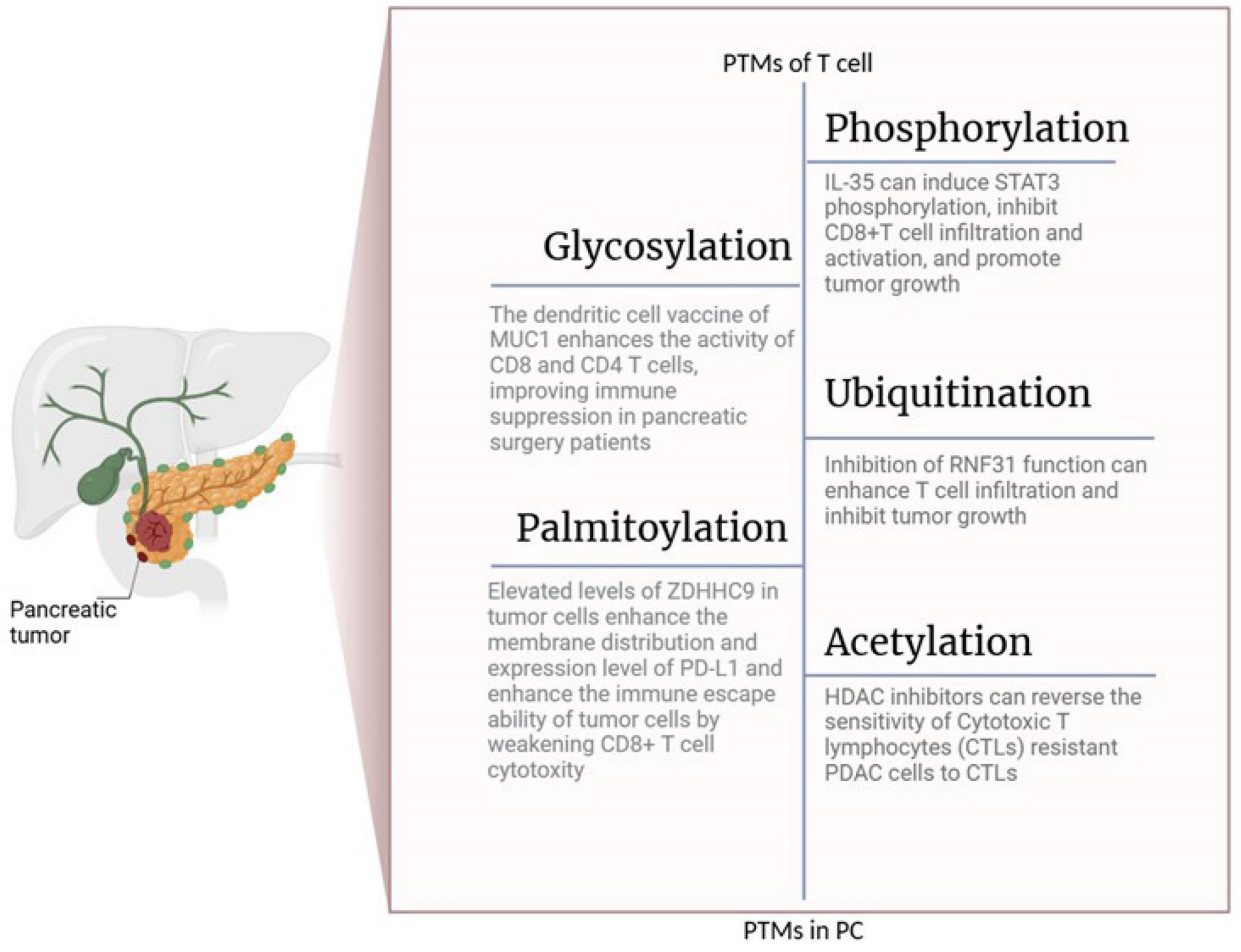
PTMs Regulate the Function of T Cells in pancreatic cancer. The figure is generated with BioRender (https://biorender.com).

Protein palmitoylation is a dynamic lipid modification facilitated by the ZDHHC protein family 134. ZDHHC9 is significantly upregulated in pancreatic cancer than in normal tissues. Elevated levels of ZDHHC9 in tumor cells enhance the membrane distribution and expression level of PD-L1 and enhance the immune escape ability of tumor cells by weakening CD8+ T cell cytotoxicity (Fig. 3) 135.

Lysine 2-hydroxyisobutyrylation (Khib) is a new PTM found in histones that primarily regulates chromatin function 136. KEGG analysis of Khib proteins reveals significant enrichment in glycolysis/gluconeogenesis pathways. Khib may significantly impact PDAC metabolism and facilitate tumor progression. The Khib protein inhibitor MG149 significantly inhibits PDAC migration and invasion, indicating that inhibitors targeting Khib proteins could be potential therapeutic targets for cancer treatment 135.

PTMs frequently interact to regulate protein functions instead of occurring independently. Emerging evidence highlights the extensive interaction among various PTMs in disease progression and treatment. O-GlcNAcylation, a PTM of SIRT7, can inhibit its interaction with REGγ and enhance histone deacetylation, consequently facilitating pancreatic cancer progression by preserving SIRT7 stability 137. Mdm2, an E3 ubiquitin ligase, is the primary negative regulator of p53, facilitating its degradation through ubiquitination 138. They possess overlapping acetylation sites, and acetylated p53 and Mdm2 repel each other, thus maintaining p53 stability and transcriptional activity 139, 140. Understanding these PTM interactions can provide valuable insights into disease mechanisms and reveal new therapeutic targets.

PTMs are essential in the regulation of protein function, stability, interactions, and subcellular localization. Understanding the role of PTMs in the immune microenvironment and immunotherapy of pancreatic cancer can improve our comprehension of the disease and aid in developing new therapeutic strategies.

## Figures and Tables

**Table 1:** Identifying phosphorylation targets associated with PDAC

Target	Function in cancer	Reference
FAM83A	Promote the transcriptional activity of β-catenin	141
PDE4D	Control the degradation of Camp.	142
Girdin	Control the cytoskeleton and vascular remodeling	143
ASPP2	Regulating cell apoptosis.	144
WAVE3	Promote epithelial mesenchymal transition and regulate metastasis.	145
IER3	Activate ERK1/2 to support the development of PanIN after pancreatitis.	146
MUC4	The transmembrane ligand of ERBB2 maintains its stability on the plasma membrane and enhances activation.	147
Stattic	Inhibition of STAT3 activation and nuclear translocation.	148
CAP1	Regulating actin cytoskeleton and cell migration.	149
CTDSPL2	Regulating mitosis and promoting cell movement.	150
IQGAP1	articipate in cytoskeleton remodeling, cell migration and intercellular signal transduction.	151

**Table 2:** Glycosylation targets associated with PDAC

Target	Function in cancer	Reference
ST3Gal1	Attaching sialic acid to T-antigen, producing sialyl T-antigen	152
MUC1	Activating the EGFR-PI3K/Akt signaling pathway and help cancer cells fight anoikis	153
CA199	Pancreatic cancer tumor biomarkers	154
MDH1	Involved in the interconversion of pyruvate and malic acid in mitochondria	155
CD44	Promoting the expression of NANOG in pancreatic cancer cells and facilitate the alteration of CSC feature	156
MGAT5	N-glycan branching through adding β1,6-linked N-acetylglucosamine (β1,6-GlcNAc) to an α1,6-linked mannose	86
TNFR1	Increased α-2,6-sialylation of TNFR1 inhibits internalization and stabilizes signaling through AKT and NF-κB, conferring resistance to gemcitabine and TNF-induced apoptosis	157

**Table 3:** Identifying acetylation targets associated with PDAC

Target	Function in cancer	Reference
P65	Deacetylation at the P65 K310 site inhibits NF-κB transcriptional activity and inhibits PD-L1 expression	97
SIRT5	SIRT5 loss enhanced glutamine and glutathione metabolism via acetylation-mediated activation of GOT1	158
HSPA5	Acetylation at K353 site of HSPA5 promoted ferroptosis of PDAC	159
BCAT2	BCAT2 acetylation suppresses BCAA catabolism and pancreatic tumor growth	160
PGC-1α	PGC-1α acetylation causes metabolism to shift from a mitochondrial oxidative catabolic process to fatty acid synthesis	161
STAT3	STAT3 acetylation inhibits the STAT3/SIRT1 interaction and enhances the function of immunosuppressive cells in pancreatic cancer	162

**Table 4:** Identifying ubiquitination and deubiquitination targets associated with PDAC

Target	Function in cancer	Reference
USP8	USP8 interacts with PD-L1 to inhibit its ubiquitination proteasome degradation	111
USP10	USP10 inhibits YAP1 ubiquitination and degradation to promote Cyr61 expression, which induces immune escape and promotes growth and metastasis of PAAD	109
USP25	USP25 regulates HIF-1α transcriptional activity and regulates metabolic reprogramming, promoting PDAC cell growth	163
USP22	USP22 deubiquitinated PD-L1 and inhibited its proteasome degradation	164
cGAS	The ubiquitination degradation of cGAS inhibited the activation of CGAS-STING signaling pathway and reduced the production of pro-inflammatory cytokines and type I interferon	165
β-catenin	Ubiquitination degradation of β-catenin leads to cell cycle arrest at G1 and promotes apoptosis	166
eEF1A1	eEF1A1 acts with FBXO32 to promote ubiquitination of eEF1A1 at K273, enhancing its activity and increasing protein synthesis in PDAC cells	167

## Abbreviations

PDAC: Pancreatic ductal adenocarcinoma
ACT: Adoptive cell therapy
CAR-T: Chimeric Antigen Receptor T-Cell
TCR-T: T-Cell Receptor-engineered T cell
TME: Tumor Microenvironment
MSI-H: Microsatellite Instability-High
PTM: Post-translational modification
SUMO: Small Ubiquitin-like Modifier
PD-1: Programmed Cell Death Protein 1
PD-L1: Programmed Death-Ligand 1
CTLA-4: Cytotoxic T-lymphocyte associated protein 4
NEK2: Never in Mitosis A-related kinase 2
GEM: Gemcitabine
STAT1: Signal Transducer and Activator of Transcription 1
Plk1: Polo-like Kinase 1
RB: retinoblastoma protein
TCR: T cell receptor TCR
TILs: tumor-infiltrating lymphocytes
TAMs: Tumor-associated macrophages
IL-35: Interleukin-35
HAS2: Hyaluronan synthase 2
DYRK1B: Dual-specificity tyrosine-regulated kinase 1B
IL-21: Interleukin-21
MUC4: Mucin 4
MUC1: Mucin 1
MGAT5: Glycosyltransferase 5
HDAC: Histone deacetylase
CTLs: Cytotoxic T lymphocytes
MDSC: Myeloid-derived suppressor cell
DUB: Deubiquitinating enzyme
USP10: Ubiquitin-specific peptidase 10
USP22: Ubiquitin-specific peptidase 22
USP8: Ubiquitin-specific peptidase 8
CSN5: COP9 signalosome subunit 5
LUBAC: linear ubiquitin chain assembly complex
Khib: Lysine 2-hydroxyisobutyrylation
